# Effects of Morphine on Serum Reproductive Hormone Levels and the Expression of Genes Involved in Fertility-related Pathways in Male Rats

**DOI:** 10.22037/ijpr.2019.112119.13544

**Published:** 2021

**Authors:** Marziyeh Ajdary, Sina Farzan, Yasaman Razavi, Abbas Arabdolatabadi, Abbas Haghparast

**Affiliations:** a *Abadan Faculty of Medical Sciences, Abadan, Iran. *; b *Cellular and Molecular Research Center, Iran University of Medical Sciences, Tehran, Iran. *; c *Department of Anesthesiology, School of Medicine, Tehran University of Medical Sciences, Tehran, Iran. *; d *Young Researchers and Elite Club, Yazd Branch, Islamic Azad University, Yazd, Iran. *; e *Neuroscience Research Center, School of Medicine, Shahid Beheshti University of Medical Sciences, Tehran, Iran.*

**Keywords:** Addiction, Morphine, Infertility pathway, Reproductive hormone, Testis

## Abstract

The effects of morphine on serum reproductive hormone levels and markers involved in fertility-related pathways were evaluated. A total of 30 male Wistar rats were divided into three groups (n = 10) and intraperitoneally administered the following substances for 20 days: two single daily doses of morphine (10 mg/kg; morphine group), saline (healthy saline), and intact group. After confirming the morphine dependence of the experimental groups, all the animals were sacrificed and their total testis tissue was extracted and stored at −80 °C until use. Male reproductive parameters (blood serum of testosterone, luteinizing hormone, and follicle-stimulating hormone) and using Q-PCR and western blot, we evaluated mRNA and protein expression of *CREM*, *TBP*, *CREB1*, *HDAC1*, and *FOS* involved in fertility-related pathways were analyzed and compared in the testis samples. The luteinizing hormone and testosterone levels were significantly lower in the morphine-administered group than in the saline and intact groups (*P *< 0.05). Moreover, the expressions of all five target genes were downregulated in the morphine group (*P *< 0.05). The protein expression of all five target proteins was downregulated in the morphine group (*P *< 0.05). We concluded that morphine could decrease the reproductive parameters in male rats.

## Introduction

Addiction to mind-altering and recreational drugs is increasingly becoming a major medical and social problem worldwide, being prevalent in rich and developing countries similarly ( 1 ). A considerable number of cases of “male factor” infertility are categorized as “idiopathic” owing to their unknown etiology ( 2 ). Infertility and problems of impaired fecundity have been a concern for ages and are also a significant clinical problem today, affecting 8–12% of couples worldwide. Approximately 40–50% of all infertility cases are due to “male factor” infertility ( 3 ).

Endogenous opioid peptides are present in different tissues of the male reproductive tract, suggesting that they may be involved in reproductive function (4). The effect of opioids on producing testosterone by inhibiting the Gonadotropin-releasing hormone (GnRH) secretion, injections of β-endorphin in the ventromedial, anterior and preoptic-septal hypothalamic areas reduce the luteinizing hormone (LH) secretion from the pituitary. To establish down-regulation of GnRH mRNA levels through morphine, an in situ hybridization was used by Li and Pelletier (5). Thus, the administration of chronic morphine inhibits the secretion of GnRH (6). On the other hand, levels of follicle-stimulating hormone (FSH) are not influenced by opioid analogs or antagonists (6). Moreover, as animal models have shown, modulation of the negative feedback of sex steroids on secreting LH can be by opioids (6). In male rats, the hypothalamus sensitivity to negative feedback is enhanced by testosterone morphine (7, 8). 

According to previous studies, morphine alone does a great deal of damage to testicular tissue (9). Salahshoor *et al*. demonstrated that addiction in the male rat adversely affected fertility (10). Fronczak *et al*. reviewed the literature on the prevalence and effects of drug abuse on male fertility (11). Studies have shown that morphine induces oxidative stress in animals by producing free radicals and ROS, thereby affecting glycolysis, the mitochondrial respiratory chain, ATP production, amino acid metabolism, the antioxidant defense system, cellular detoxification, and gene toxicity (12). 

There is a possible association between the increased side effect of addiction and gene expression changes; therefore, ways of reversing or modifying the drug action may be provided by finding genes whose expression possibly will underlie these phenomena. Also, there are many reports about other changes in gene expression induced by morphine (13, 14); but, changes in gene expression in the testis upon addiction to drugs abused are rarely known. In this study, to select a gene, we selected the entire genes associated with male infertility through the NCBI Entrez-Gene database using the keywords “male infertility”, “spermatogenesis”. The results showed that *CREM, TBP, CREB1, HDAC1*, and *FOS* had the most relationship with male infertility. In this study, we studied the effect of morphine toxicity on these genes. Chronic morphine treatment was found to change the expression of FBJ osteosarcoma oncogene (*FOS*) and cAMP-responsive element-binding protein 1 (*CREB1*), cAMP-responsive element modulator (*CREM*) (13) in the striatum and nucleus accumbens (13) it also increases the degeneration tissue, cytokines, stress signals such as free oxygen radicals (14), mitogenic stimulation (15), infections, and oncogenic compounds (16) and oxidative stress (17); also, it decreases growth factor in the various organs of rats (18). Another study revealed opioids’ effect on the expression of histone deacetylase 1 (*HDAC1*) and inducing tumor necrosis in the rat (19); also,* HDAC1* -located in the nucleus of Sertoli cells of the testis- is involved in the spermatogenesis pathway (20). A series of experiments showed that the lack of expression of TATA-box-binding protein (*TBP*) suppressed the transcription of RNA polymerase III during mitosis (21). Given the potential adverse effects of morphine on male rat fertility, we aimed to determine the expression of these target genes involved in fertility-related pathways in opiate-addicted male rats, as well as the serum reproductive hormone levels.

## Experimental

*Animals*

For this study, 30 male Wistar rats (200–250 g) were purchased from Tehran Pasteur Institute and kept in standard cages at the animal house of the Iran University of Medical Sciences. Before the experiments, the animals were fed a normal diet and water to acclimatize them to the environment and establish physiological adaptation. Throughout the study, the rats were kept under similar conditions, at a temperature of 22 ± 2 °C with 12 h light and 12 h darkness and free access to water and food. All experiments were conducted according to the guide for the care and use of laboratory animals, and were approved by the research and ethics committee of the Abadan University of Medical Sciences (IR-ABDANUMS.1395.121).

*Chemicals*

Morphine (C_16_H_19_NO_3_) was obtained from TEMAD Chemical Company (Tehran, Iran) and naloxone hydrochloride from Darou Pakhsh (Tehran, Iran). Both drugs were dissolved in saline (0.9%) for administration (22).

*Experimental protocol*

Rats were divided into three groups (n = 10): morphine group, saline and control groups. 

*Morphine administration*

Morphine was freshly dissolved in 0.9% saline solution before each administration. Rats received repeated injections of the same dose (10 mg/kg [sc], twice/day, for 20 consecutive days). The saline group received a subcutaneous (S.C) injection of saline (1 mL/kg) at the same volume twice daily for 20 consecutive days. in the last control groups, the animals didn’t receive any drugs. Injections were performed at 09:00 and 15:00.

To determine the best protocol for the induction of addiction to morphine, a single dose of morphine (10 mg/kg) was used to determine its magnitude. In this set of experiments, rats received a dose of morphine (10 mg/kg [sc]) twice/day for 20 days. After that, On the twentieth day, naloxone (1 mL/kg) was injected 30 min after last morphine injection and morphine withdrawal signs were monitored (23, 24). The withdrawal symptoms included tremors and movement of the limbs off the baseline floor.

*Hormone assay*

Blood serum was separated from the collected blood by centrifugation (4000 ×*g *for 10 min). The serum samples were stored in a deep freezer at −20 °C. The blood testosterone, LH, and FSH concentrations were measured by enzyme-linked immunosorbent assays (Abcam 108666, Cambridge, MA, USA) (4).

*RNA extraction and real-time quantitative reverse-transcription PCR*

Total RNAs from the testis tissue ere extracted and purified with TRIzol reagent (Sigma, Pool, UK), following themanufacturer’s instructions. The RNA concentration was measured with a NanoDrop ND-100 spectrophotometer. The reverse transcription-polymerase chain reaction (RT*-*PCR) was performed to monitor the gene expression levels of *HDAC1*, *CREM*, *TBP*, *FOS*, and *CREB1*. A 500-ng sample of RNA was reverse transcribed with the Transcriptor High Fidelity cDNA Synthesis Kit (Invitrogen, Paisley, UK), using oligo(dT) primers (Roche, Basel, Switzerland). One microliter of the cDNA was amplified using the Opticon II system (Invitrogen, Paisley, UK) and the SYBR Green PCR Master Mix (Invitrogen, Paisley, UK), following the manufacturer’s instructions. Forty PCR cycles were performed, using an annealing temperature of 60 °C for all the genes tested. Primers were specifically designed between two adjacent exons (Gene Runner program); the sequences used in this study are presented in Table 1. The mRNA levels of these genes (Ct) were normalized to that of the reference gene glyceraldehyde-3-phosphate dehydrogenase (*GAPDH*) by subtracting the Ct value of *GAPDH* from the Ct value of the sample (ΔCt = CT_ Sample _– CT_ Reference_). The relative expression of the target gene to the calibrator was quantified using 2^-ΔΔCt^. 

*Western Blot (WB)*

Based on materials,criteria WB was performed as described previously (25). In brief, after testis removal, complete testis was bilaterally micro dissected on ice, snap frozen and stored at −80 °C. Samples were homogenized with complete protease inhibitor cocktail (Roche, Mannheim, Germany), centrifuged and protein levels were measured according to Bicinchoninic Acid (BCA) protein assay method (Sigma-Aldrich). An equal amount of total protein (30 mg) was resolved on SDS-PAGE gels (8–10%) and transferred to polyvinylidene fluoride (PVDF) membranes via electrophoretic transfer system (Bio-Rad, Munchen, Germany). The membranes were blocked and incubated with specific primary antibodies at 4 °C overnight. Primary antibodies were rat monoclonal antibodies to HDAC1 (1:500, Santa Cruz Biotechnology), *CREM* (1:500, Santa Cruz Biotechnology), *TBP* (1:500, Santa Cruz Biotechnology), *FOS* (1:500, Santa Cruz Biotechnology), *CREB1*(1:500, Santa Cruz Biotechnology) as well as mouse monoclonal antibodies to *GAPDH* (1:500, Santa Cruz Biotechnology) to monitor loading. The membranes were washed with PBS, 0.05% Tween-20 (PBS-T), and incubated with respective HRP conjugated secondary antibodies (1:1000) at 4 °C for 4 h. The blots were exposed to HRP substrate solution (3, 3′-Diaminobenzidine and H_2_O_2_) for detection of target antigens. After staining, bands intensity was quantified using ImageJ (http://rsb.info.nih.gov/ij/) software after background subtraction and band density normalization.

*Statistical analysis*

The normal distribution of the data was evaluated using the Kolmogorov-Smirnov test. All data are presented as the mean ± SEM. The statistical significance of differences between the groups was determined using one-way analysis of variance, followed by Tukey’s *post-hoc* test. Statistical Package for the Social Sciences software (version 16.0, SPSS Inc., Chicago, IL, USA) was used for all the analyses. A value of *P* < 0.05 was considered statistically significant.

## Results

*Effects of morphine on serum reproductive hormone levels*

**Figure 1:**
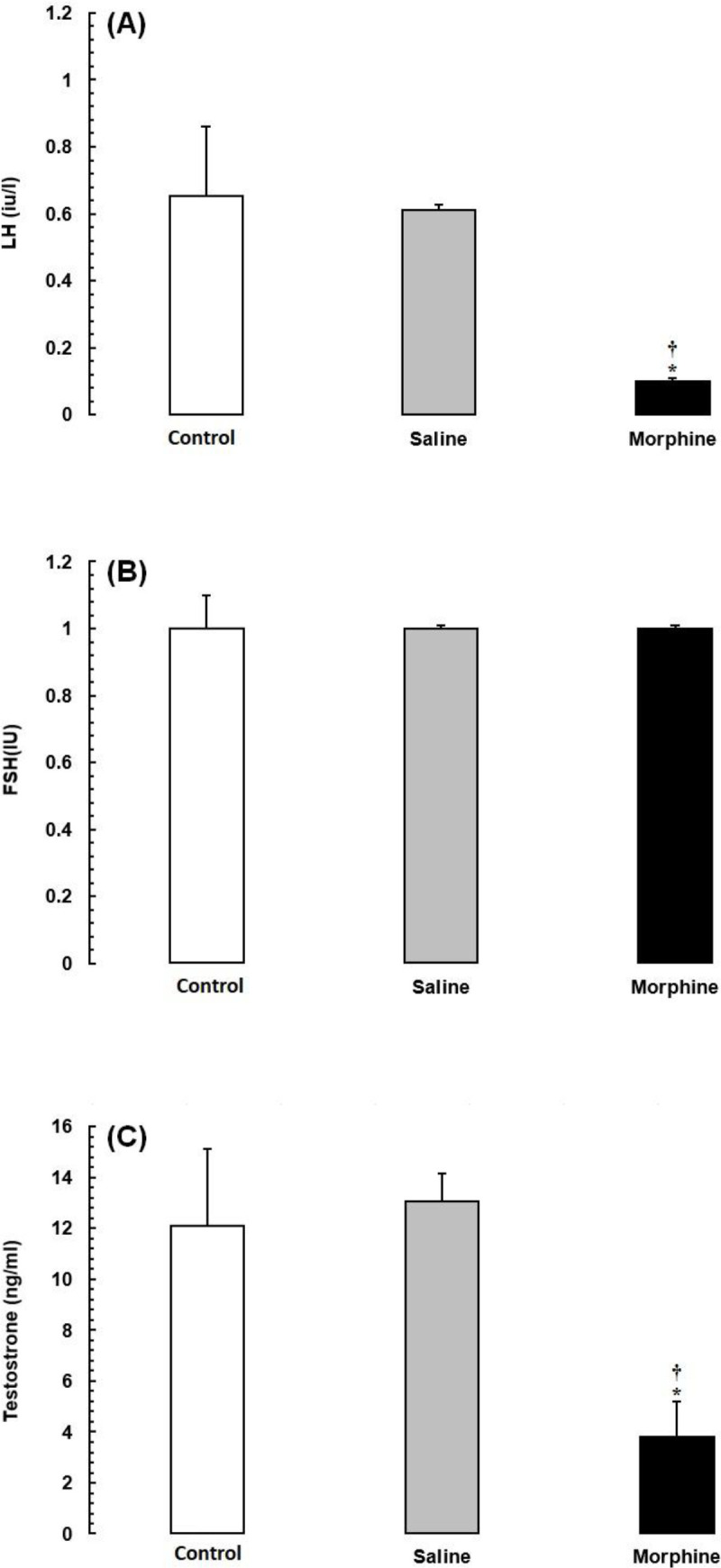
Effects of morphine on serum reproductive hormone levels in Wistar rats. Data are expressed as the mean ± SEM; ^*^Significant decrease of the hormone level in the morphine group compared with the saline and control groups (*P* < 0.05).

As shown in Figure 1, the mean serum levels of LH and testosterone were significantly lower in the opiate-addicted subjects than in animals of the control and saline groups (*P* < 0.05). The FSH levels were also lower in the morphine group, although the difference was not statistically significant (*P *> 0.05).

*Effects of morphine on genes of the male fertility-related pathways*

**Figure 2:**
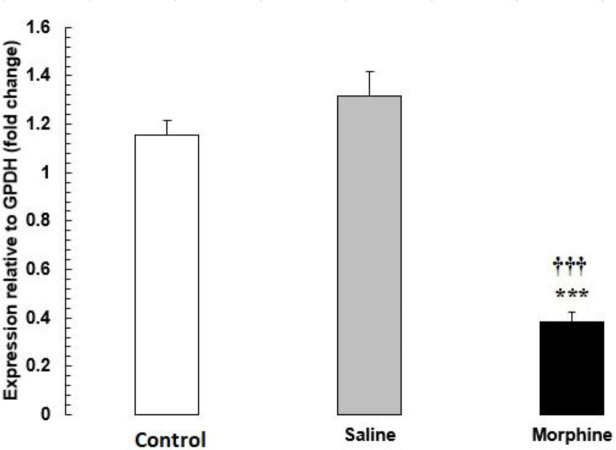
*HDAC1 *mRNA expression in testis tissue from male rats addicted to morphine. The mRNA amounts were evaluated by a quantitative real-time reverse-transcription polymerase chain reaction. Data are the mean ± SEM (n = 10 for each group). Glyceraldehyde-3-phosphate dehydrogenase was used as an internal control. ^***^P < 0.001, morphine group *vs. *saline and control groups

The mRNA levels of *HDAC1* were significantly lower in the morphine group than in the saline and control groups ( *P* < 0.001) ( Figure 2 ).

**Figure 3:**
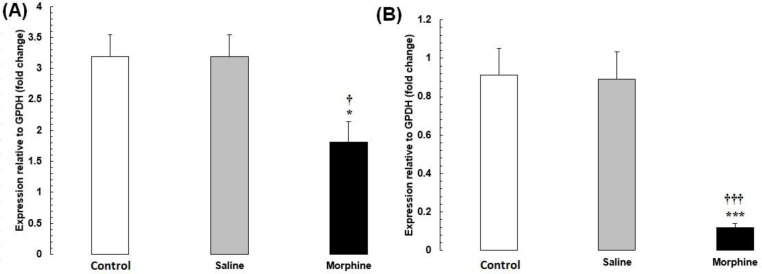
(A) *CREM* and (B) *TBP* mRNA expression in testis tissues from male rats addicted to morphine. The mRNA amounts were evaluated by a quantitative real-time reverse-transcription polymerase chain reaction. Data are the mean ± SEM (n = 10 for each group). Glyceraldehyde-3-phosphate dehydrogenase was used as an internal control. ^*^P < 0.05 and ^***^P < 0.001, morphine group *vs. *saline and control groups

The mRNA levels of *CREM* and *TBP* were significantly lower in the morphine group than in the saline and control groups ( *P* < 0.001) ( Figure 3 ).

**Figure 4:**
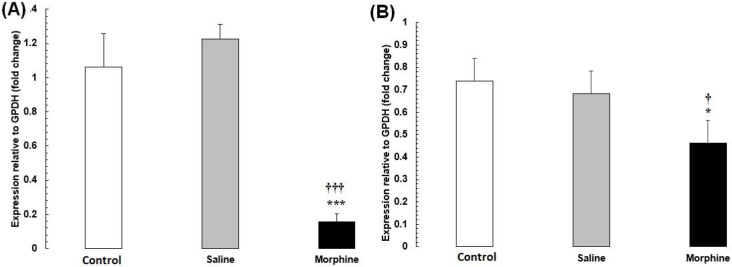
(A) *CREB1* and (B) *FOS* mRNA expression in testis tissues from male rats addicted to morphine. The mRNA amounts were evaluated by a quantitative real-time reverse-transcription polymerase chain reaction. Data are the mean ± SEM (n = 10 for each group). Glyceraldehyde-3-phosphate dehydrogenase was used as an internal control. ^*^P < 0.05 and ^***^P < 0.001, morphine group *vs. *saline and control groups

The mRNA levels of *CREB1* and *FOS* were significantly lower in the morphine group than in the saline and control groups ( *P* < 0.001) ( Figure 4 ).

*Confirmation of PCR-real time results by western blot analysis*

**Figure 5.**
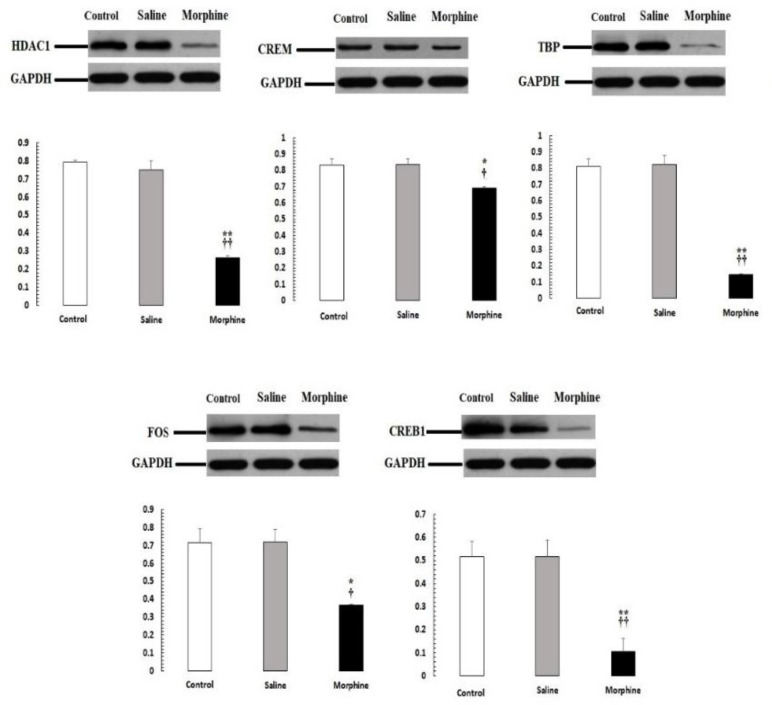
*CREM*, *TBP*, *CREB1*, *HDAC1*, and *FOS* protein levels were evaluated by Western blot. Data are the mean ± SEM (n = 10 for each group). Glyceraldehyde-3-phosphate dehydrogenase was used as an internal control. ^*^P < 0.05 and ^**^P < 0.01, morphine group *vs. *saline and control groups

Our PCR-real time results were confirmed by western blot analysis of all proteins, including *HDAC1, CREM, TBP, CREB1, FOS* (Figure 5). The expression of these proteins was significantly down-regulated (*P* < 0.05) in male rats with addict compared with control and saline. 

## Discussion

Our study detected significant associations of opiate addiction with the impairment of male fertility-related parameters and decreased markers in fertility-related pathways. Besides, the serum LH and testosterone levels were significantly lower in the addicted rats than in the saline and control rats. Despite the presence of hypotestosteronemia, low LH, and impaired spermatogenesis, the serum level of FSH did not increase in the addicted rats. These results are consistent with previous studies demonstrating that opiate consumption can result in a state of hypogonadotropic hypogonadism (26). In another similar study, it was shown that the administration of morphine to male rats led to a decrease in serum levels of LH and testosterone and decreases in the testis weight and sperm production (27). A subsequent study by Lee *et al.* in 1978 on addicts confirmed the lowering of the blood testosterone levels, but changes in the FSH and LH levels were not determined at that time (28). In animal studies conducted on rats and pigs, there was no change in the FSH levels despite a significant decrease in the LH and testosterone levels (29). Drug use appears to change the sexual function and the level of associated hormones (29). A decline in male fertility has occurred in recent years, with one of the main reasons for increased exposure to toxicants in the environment. These agents may be chemical materials, stress, and ionizing radiation, as well as substance abuse (30, 31). In line with our data, this study showed that administration of morphine also reduced serum reproductive hormones and confirmed that the damage induced by morphine caused to spermatogenesis.

Since previous studies have investigated the effect of opioids on male infertility, they examined various molecular pathways in testicular tissues. They showed that drug use was effective on pathways involved in infertility. In our present study, the morphine group had a significantly lower expression of male fertility genes. In addition, this finding implies that the impaired male fertility found in some cases could be due to drug influences in the hypothalamus and pituitary gland anda direct effect on the DNA integrity in testis tissue. The genes and proteins related to spermatogenesis and metabolism can be effective in normal sperm function. One of these genes is *HDAC1*, which is present in the nucleus of the Sertoli cells responsible for producing sperm. On the other hand, opiates have receptors in different parts of the testicles, including Sertoli cells and seminiferous tubules, which confirms their direct impact on spermatogenesis. They affect the *HDAC1* gene in the nucleus of Sertoli cells and disrupt the spermatogenesis cycle; this gene has a critical role in regulating the changes in the major histones and in the control of chromatin changes that affect the transcription of tumor suppressor genes, causing tumorous and pathological changes in the tissue (20). *HDAC1* is linked to different pathways, including the mTOR, MAPK, Notch, Hippo and Wnt signaling pathways associated with cell growth, proliferation and differentiation (32).

Different studies have shown that disruption in each of these pathways is associated with infertility in both men and women (33, 34). A study conducted by Jee Hyun Kim in 2014 showed that the level of *HDAC1* expression in men with azoospermia was lower than that of normal people and attributed it to the reduction of *HDAC1* gene expression with sperm DFI (35). 

The assessment of *CREB, FOS* in our study showed that its protein expression was diminished in the addict animal which is in agreement with a previous research Elena H. Chartoff *et al*. suggested that decrease *CREB1, FOS* activation in portions of the striatum is related to addict by morphine (36). The gene transcript for CBP that is a protein binds to *CREB* (CBP or Crebbp) and strongly linked protein p300 are essential cofactors for many nuclear transcription factors (37). Since direct pathways for CBP activation by GnRH and insulin, *i.e.,* mitogen-activated protein kinase (MAPK) and PKC can phosphorylate CBP, CBP action in the gonadotroph was studied (38). As FSH increases, it affects the Sertoli receptors and increases the adenylate cyclase and cAMP levels, resulting in phosphorylation of the *CREB1* transcription factor on serine-133 (39). *CREB1* induces the transcription factors required to activate other genes involved in spermatogenesis (40). It regulates the hormones that regulate spermatogenesis and anti-apoptotic factors (41). According to the data shown by the String database, there is an interaction between *FOS* with Interleukins receptor, apoptosis-related cysteine peptidase (CASP1), Mitogen-activated protein kinase 14 (MAPK14), Jun proto-oncogene (JUN), and FBJ murine (42); in addition, according to another study, in evaluating specific types of male infertility, Interleukins and *FOS* in seminal plasma should be extended; Thus, the changed *FOS* influences the innate immunity level in male infertility (43). 

Studies have shown that *CREB1, CREM, *and* FOS* in the mouse and rat spermatogonial stem cell model helps the process of spermatogenesis by activating the Ras/Erk1/2 pathway and CDK2 promoter (44, 45). For expressing the late spermatogenic genes, there is a known master switch, including several spermatid-specific transcriptional regulators. The transcriptional activator CREM_T_, highly expressed in round spermatids is essential for expressing many important postmeiotic genes, for instance, *Prm1*, *Prm2*, *Tnp1* and *Tnp2, is *encoded by the *Crem* gene (46, 47).

The coordinated action of a set of general transcription factors is necessary for transcription initiation by RNA polymerase II in eukaryotes (48, 49). The *TBP* is one of the major factors in transcription initiation; as well, during spermatogenesis, it shows a stage-specific expression pattern (50). Previous studies showed that testicular tissue in sterilized male mice was investigated, and the number of genes involved in spermatogenesis. The number of sperms in these mice was also evaluated. This study showed that spermatozoa were decreased and spermatogenesis was controlled by the *TBP* gene and *TBP* has been shown to affect the various factors involved in spermatogenesis (51). 

Vesselin M. Chorbov et al. (2011) showed that DNA methylation in addicted men’s sperm was higher than those who quit the addiction. Hypermethylation of CpG loci in the promoter of genes involved in suppressing necrotic tumors, such as HDAC1 and HDAC1 gene expression, decreases. Increased DNA methylation in sperm may represent a method of epigenetic inheritance of opioid abuse or dependence phenotypes (52). According to Betina González in 2018, drug addiction modifies epigenetic homeostasis and next-generation outcomes, increases methylation of cytosine levels in sperm DNA and germ cells, and decreases HDAC1 gene expression. It also decreases spermatogenesis, which has a higher gene expression rate with drug withdrawal but is lower than normal (53). Jinghua Wang (2007) stated that morphine chronically inhibits interleukin-2 (IL-2) at the genomic and protein levelresulting in the inhibition of the CREB gene. In addition, chronic morphine treatment inhibits acetylation and trimethylation of histones and decreases DNA demethylation and access to the IL-2 promoter. These findings suggest that chronic morphine treatment may act through both transcriptional and epigenetic mechanisms to inhibit the IL-2 production and ultimately reduce CREB gene expression (13). Alcohol, cocaine, and nicotine increase methylation of cytosine levels in DNA and decrease FOS gene expression (54). According to another study, cellular and molecular examination in mice showed that spermatogonia stem cells were necessary for sperm production. Also, knocking down a set of genes, especially the *TBP* gene, reduces the production of sperm, which also increases apoptosis rates in these rats (55).

**Table 1:** Sequence of primers used in the current investigation

**Gene**		**Sequence (5'->3')**	**Length**	**Tm**	**GC (%)**	**Self-complementarity**	**Self-3' complementarity**
*HDAC1*	Forward primer	GACGGGGATGATGGAAACTAC	21	58.16	52.38	3.00	0.00
	Reverse primer	GTTGGATTTGTGAGGACGATAG	22	56.87	45.45	2.00	2.00
*CREM*	Forward primer	GAAACAACATAGGGTAGAAAGGG	23	56.85	43.48	2.00	0.00
	Reverse primer	GAAAATGAGCACAACACTGGATG	23	58.77	43.48	4.00	1.00
*TBP*	Forward primer	ATCTTCATCCTTGTCCTCCAGCTTC	25	62.38	48.00	4.00	0.00
	Reverse primer	GCTCCCTCCAAAGCAATCTTCCTTA	25	62.85	48.00	6.00	4.00
*FOS*	Forward primer	GGTCCTGTCTGGTTCCTTCTATG	23	60.12	52.17	3.00	0.00
	Reverse primer	CTGCCTTGTCTGACTGCTCAC	21	61.21	57.14	5.00	1.00
*CREB1*	Forward primer	CAG TTG TTA TGG CGTCCT	18	54.61	50.00	2.00	2.00
	Reverse primer	CTT GCT GCT TCC CTG TTC	18	55.99	55.56	3.00	0.00
*GAPDH*	Forward primer	CAT ACT CAG CAC CAG CAT CAC C	22	61.32	54.55	3.00	0.00
	Reverse primer	AAG TTC AAC GGC ACA GTC AAG G	22	61.58	50.00	5.00	0.00

## Conclusion

In conclusion, we have indicated for the first time the adverse effects of morphine on key molecules involved in fertility-related pathways in the male rat. It appears that morphine could decrease the reproductive parameters in this animal model of addiction. We suggest that morphine was able to affect the downregulation of the key molecule in the fertility-related pathways in the male rat. 
